# Comparisons of Damage Evolution between 2D C/SiC and SiC/SiC Ceramic-Matrix Composites under Tension-Tension Cyclic Fatigue Loading at Room and Elevated Temperatures

**DOI:** 10.3390/ma9100844

**Published:** 2016-10-19

**Authors:** Longbiao Li

**Affiliations:** College of Civil Aviation, Nanjing University of Aeronautics and Astronautics, No. 29, Yudao St., Nanjing 210016, China; llb451@nuaa.edu.cn

**Keywords:** ceramic-matrix composites (CMCs), fatigue, damage evolution

## Abstract

In this paper, comparisons of damage evolution between 2D C/SiC and SiC/SiC ceramic-matrix composites (CMCs) under tension–tension cyclic fatigue loading at room and elevated temperatures have been investigated. Fatigue hysteresis loops models considering multiple matrix cracking modes in 2D CMCs have been developed based on the damage mechanism of fiber sliding relative to the matrix in the interface debonded region. The relationships between the fatigue hysteresis loops, fatigue hysteresis dissipated energy, fatigue peak stress, matrix multiple cracking modes, and interface shear stress have been established. The effects of fiber volume fraction, fatigue peak stress and matrix cracking mode proportion on fatigue hysteresis dissipated energy and interface debonding and sliding have been analyzed. The experimental fatigue hysteresis dissipated energy of 2D C/SiC and SiC/SiC composites at room temperature, 550 °C, 800 °C, and 1100 °C in air, and 1200 °C in vacuum corresponding to different fatigue peak stresses and cycle numbers have been analyzed. The interface shear stress degradation rate has been obtained through comparing the experimental fatigue hysteresis dissipated energy with theoretical values. Fatigue damage evolution in C/SiC and SiC/SiC composites has been compared using damage parameters of fatigue hysteresis dissipated energy and interface shear stress degradation rate. It was found that the interface shear stress degradation rate increases at elevated temperature in air compared with that at room temperature, decreases with increasing loading frequency at room temperature, and increases with increasing fatigue peak stress at room and elevated temperatures.

## 1. Introduction

Ceramic materials possess high strength and high modulus at elevated temperatures. However, their use as structural components is severely limited due to the brittleness. Continuous fiber-reinforced ceramic-matrix composites (CMCs), are fabricated by incorporating fibers into ceramic matrices, and provide an alternative to conventional ceramics in high temperature applications. CMCs retain the strength, low weight, and high temperature capability while exhibiting less brittleness than ceramics. The CMCs exceed the capability of current nickel super alloys used in high-pressure turbines and provide increased efficiency [1].

The CMCs are subject to fatigue upon cyclic mechanical loads at fixed temperature with both room temperature and elevated temperature examined [2]. Besides, at intermediate temperatures, the chemical attack would cause damage inside of CMCs at constant mechanical load [3]; and creep degradation would occur in CMCs under constant mechanical load and constant temperature [4]. Understanding the damage mechanisms of fatigue represents an important step in the engineering applications of these materials. For 2D woven C/SiC composite, the fatigue limit stress of 100,000 cycles approaches to 90% tensile strength at room temperature [5]. Under cyclic fatigue loading, the fatigue damage mechanism of matrix multicracking, interface debonding and slipping, and interface wear occur inside of 2D C/SiC composite, leading to more fibers pullout compared with tensile specimen observed in the fracture surface. However, the fatigue life and modulus degradation were affected by the loading frequency [6,7]. When the loading frequency increases to 375 Hz, the fatigue life of 2D C/SiC composite greatly decreases due to localized oxidation at fibers surface caused by internal frictional heating [7]. The post fatigue tensile strength increased after fatigue loading due to a decrease of stress concentrations present near the crossover points of the longitudinal and transverse yarns. At 550 °C in air, there was an increase in cycle to failure at a given stress level when the loading frequency increased from 0.1 to 375 Hz [8], which is different from the trend at room temperature [7]. The oxidation of carbon fibers was almost absent at the high loading frequency of 375 Hz at 550 °C due to internal frictional heating, which caused no reduction in cycles to failure at elevated temperature in comparison to the counterparts at room temperature. At 1200 °C in vacuum, the apparent ratcheting phenomenon was highly relevant to the fatigue behavior of 2D C/SiC composite [9]. For 2D SiC/SiC composite, the fatigue limit stress at 1000 °C in argon is much lower than that at room temperature [10]. At 800 °C in air condition, the stress-strain hysteresis loops at different fatigue cycle numbers, modulus variation over fatigue cycle, cycle history of maximum strain, minimum strain and the width of stress-strain loops, changed with applied cycles [11]. At 1100 °C in air condition, the fatigue life S–N curves for the notched and unnotched specimens were quite similar, however, the fatigue strength of the notched specimens was 10% to 15% less than the unnotched fatigue strength [12]. Under cyclic fatigue loading, the stress-strain hysteresis loops are an effective tool to indicate the fatigue damage mechanisms of CMCs through analyzing fatigue hysteresis modulus or fatigue hysteresis loops area [13,14,15,16]. The fiber/matrix interface shear stress, which transfers load between fibers and matrix and affects the damage evolution in CMCs, can be obtained through hysteresis dissipated energy [17,18]. In cross-ply and 2D woven CMCs, the matrix cracking modes involve matrix cracking and interface debonding in the longitudinal plies or yarns occurs under cyclic loading [19,20]. It should be noted that the fatigue damage mechanisms and fatigue life S–N curves of cross-ply or 2D C/SiC and SiC/SiC composites have been analyzed [5,6,7,8,9,10,11,12,13,14,15,16,17,18,19,20,21]. However, the comparisons of fatigue damage evolution versus cycle numbers between C/SiC and SiC/SiC composites using damage parameters of fatigue hysteresis dissipated energy and interface shear stress have not been analyzed, which is important for the real applications on aero engine components.

The objective of this paper is to compare the damage evolution in 2D C/SiC and SiC/SiC composites under tension–tension cyclic fatigue loading at room and elevated temperatures. The fatigue hysteresis loops models considering multiple matrix cracking modes in 2D CMCs have been developed based on the damage mechanism of fiber sliding relative to matrix in the interface debonded region. The relationships between fatigue hysteresis loops, fatigue hysteresis dissipated energy, fatigue peak stress, matrix multiple cracking modes, and interface shear stress have been established. The effects of fiber volume fraction, fatigue peak stress and matrix cracking mode proportion on fatigue hysteresis dissipated energy and interface debonding and slipping have been analyzed. The experimental fatigue hysteresis dissipated energy of 2D C/SiC and SiC/SiC composites at room temperature, 550 °C, 800 °C, and 1100 °C in air, and 1200 °C in vacuum corresponding to different fatigue peak stresses and cycle numbers have been analyzed. The interface shear stress degradation rate has been obtained through comparing the experimental fatigue hysteresis dissipated energy with theoretical computational values. The damage evolution in 2D C/SiC and SiC/SiC composites have been compared using damage parameters of fatigue hysteresis dissipated energy and interface shear stress degradation rate.

## 2. Hysteresis Theories

**Figure 1:**
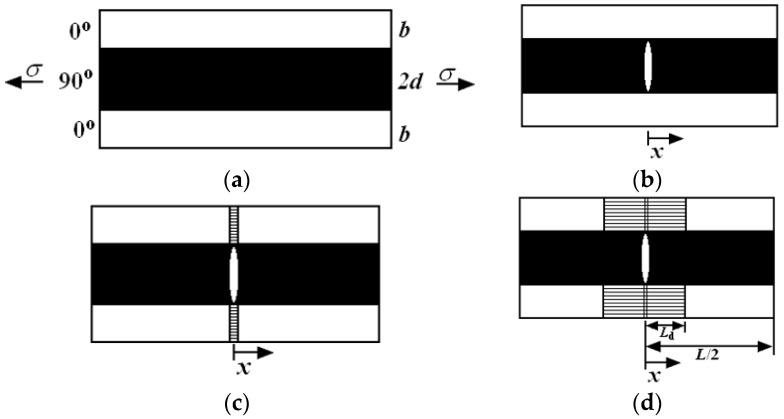

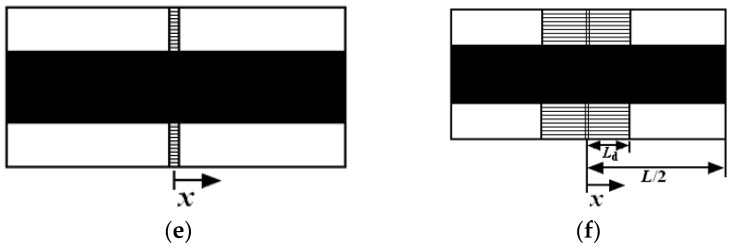
The undamaged state and five damaged modes of cross-ply and 2D woven ceramic composites: (**a**) undamaged composite; (**b**) mode 1: transverse cracking in the transverse tow, with debonding at tow boundary; (**c**) mode 2: transverse cracking and matrix cracking with perfect fiber/matrix bonding, and fracture of fibers occurs in the longitudinal tow; (**d**) mode 3: transverse cracking and matrix cracking with fiber/matrix debonding and sliding in the longitudinal tow; (**e**) mode 4: matrix cracking with perfect fiber/matrix bonding, and fracture of fibers occurs in the longitudinal tow; and (**f**) mode 5: matrix cracking and fiber/matrix interface debonding and sliding in the longitudinal tow [22].

Upon first loading to fatigue peak stress *σ*_max_, which is greater than the initial cracking stress of transverse and longitudinal ply or yarn, it is assumed that transverse cracks and matrix cracks would extend throughout the entire laminate cross-section. The multicracking modes in the cross-ply or 2D woven CMCs can be classified into five different modes, as shown in Figure 1, including: [22]

1. Cracking mode 1: Transverse cracking in the transverse tow, with debonding at tow boundary.
2. Cracking mode 2: Transverse cracking and matrix cracking with perfect fiber/matrix bonding, and fracture of fibers occurs in the longitudinal tow.
3. Cracking mode 3: Transverse cracking and matrix cracking with fiber/matrix debonding and sliding in the longitudinal tow.
4. Cracking mode 4: Matrix cracking with perfect fiber/matrix bonding, and fracture of fibers occurs in the longitudinal tow.
5. Cracking mode 5: Matrix cracking and fiber/matrix interface debonding and sliding in the longitudinal tow.

**Figure 2:**
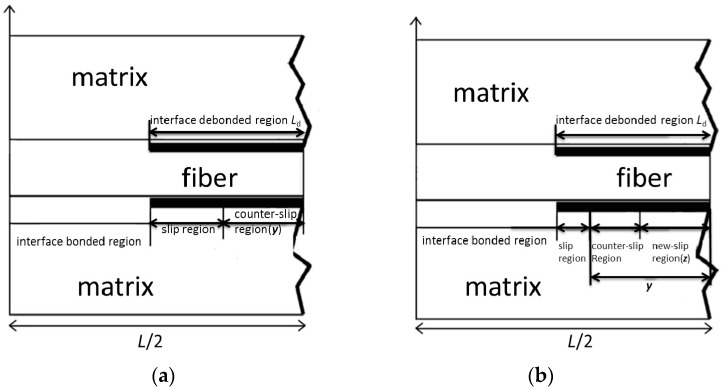
The schematic figure for fiber slipping relative to matrix upon: (**a**) unloading; and (**b**) reloading [18].

The hysteresis loops develop as a result of energy dissipation through frictional sliding between fibers and the matrix upon unloading and subsequent reloading. In the matrix cracking modes mentioned above, the interface debonding and sliding occur in the matrix cracking mode 3 and mode 5. The shape, location and area of hysteresis loops of 2D woven CMCs depend on the interface debonding and sliding in cracking in cracking mode 3 and mode 5. The schematic figure for fiber sliding relative to matrix upon unloading and reloading is illustrated in Figure 2 [18]. A unit cell is extracted from the CMCs, which contains a single fiber surrounded by a hollow cylinder of matrix. The fiber radius is *r*_f_, and the matrix radius is *R* (*R* = *r*_f_/*V*_f_^1/2^). The length of the unit cell is *L*/2, which is half matrix crack spacing, and the interface debonded length is *L*_d_. Upon unloading, counter slip occurs in the interface debonded region. The interface debonded region can be divided into two regions, i.e., interface counter-slip region and interface slip region, as shown in Figure 2a. The interface counter-slip length is denoted to be *y*. Upon reloading, new slip occurs in the interface debonded region. The interface debonded region can be divided into three regions, i.e., interface new-slip region, interface counter-slip region, and interface slip region, as shown in Figure 2b. The interface new-slip region is denoted to be *z*. Based on the damage mechanism of fiber sliding relative to matrix upon unloading and subsequent reloading, the hysteresis loops can be divided into four different cases, including:

1. Case 1: Interface partially debonds, and fiber completely slides relative to matrix.
2. Case 2: Interface partially debonds, and fiber partially slides relative to matrix.
3. Case 3: Interface completely debonds, and fiber partially slides relative to matrix.
4. Case 4: Interface completely debonds, and fiber completely slides relative to matrix.

### 2.1. Matrix Cracking Mode 3

The unloading strain *ɛ*_cu_ and reloading strain *ɛ*_cr_ corresponding to the interface slip Case 1 and Case 2 are determined by Equations (1) and (2), respectively [23].

(1) $$\varepsilon_{\mathrm{cu}}=\frac{\sigma }{V_{f\_\mathrm{axial}}E_{f}}+4\frac{\tau_{i}}{E_{f}}\frac{y^{2}}{r_{f}L}-2\frac{\tau_{i}}{E_{f}}\frac{\left(2y-L_{d}\right)\left(2y-L+L_{d}\right)}{r_{f}L}-\left(\alpha_{c}-\alpha_{f}\right)\Delta Τ$$

(2) $$\begin{array}{l} \varepsilon_{\mathrm{cr}}=\frac{\sigma }{V_{f\_\mathrm{axial}}E_{f}}-4\frac{\tau_{i}}{E_{f}}\frac{z^{2}}{r_{f}L}+\frac{4\tau_{i}}{E_{f}}\frac{{\left(y-2z\right)}^{2}}{r_{f}L}\\ \;+2\frac{\tau_{i}}{E_{f}}\frac{\left(L_{d}-2y+2z\right)\left(L_{d}+2y-2z-L\right)}{r_{f}L}-\left(\alpha_{c}-\alpha_{f}\right)\Delta Τ \end{array}$$

where *V*_f_axial_ denotes the fiber volume fraction along the loading direction; *E*_f_ denotes the fiber elastic modulus; *τ*_i_ denotes the interface shear stress; *L* denotes the matrix cracking space; *L*_d_ denotes the interface debonded length; *y* and *z* denotes the interface counter-slip and new-slip length, respectively; *r*_f_ denotes the fiber radius; *α*_f_, and *α*_c_ denote the fiber and composite thermal expansion coefficient, respectively; and Δ*T* denotes the temperature difference between the fabricated temperature *T*_0_ and testing temperature *T*_1_ (Δ*T* = T_1_ − *T*_0_).

The unloading strain *ɛ*_cu_ and reloading strain *ɛ*_cr_ corresponding to interface slip Case 3 and Case 4 are determined by Equations (3) and (4), respectively [23].

(3) $$\varepsilon_{\mathrm{cu}}=\frac{\sigma }{V_{f\_\mathrm{axial}}E_{f}}+4\frac{\tau_{i}}{E_{f}}\frac{y^{2}}{r_{f}L}-2\frac{\tau_{i}}{E_{f}}\frac{{\left(2y-L/2\right)}^{2}}{r_{f}L}-\left(\alpha_{c}-\alpha_{f}\right)\Delta Τ$$

(4) $$\varepsilon_{\mathrm{cr}}=\frac{\sigma }{V_{f\_\mathrm{axial}}E_{f}}-4\frac{\tau_{i}}{E_{f}}\frac{z^{2}}{r_{f}L}+4\frac{\tau_{i}}{E_{f}}\frac{{\left(y-2z\right)}^{2}}{r_{f}L}-2\frac{\tau_{i}}{E_{f}}\frac{{\left(L/2-2y+2z\right)}^{2}}{r_{f}L}-\left(\alpha_{c}-\alpha_{f}\right)\Delta Τ$$

### 2.2. Matrix Cracking Mode 5

The unloading strain *ε*_cu_ and reloading strain *ε*_cr_ corresponding to interface slip Case 1 and Case 2 are determined by Equations (5) and (6), respectively [23].

(5) $$\varepsilon_{\mathrm{cu}}=\frac{1}{V_{f\_\mathrm{axial}}E_{f}}\left(\sigma -k\sigma_{\mathrm{to}}\right)+4\frac{\tau_{i}}{E_{f}}\frac{y^{2}}{r_{f}L}-2\frac{\tau_{i}}{E_{f}}\frac{\left(2y-L_{d}\right)\left(2y+L_{d}-L\right)}{r_{f}L}-\left(\alpha_{c}-\alpha_{f}\right)\Delta Τ$$

(6) $$\begin{array}{l} \varepsilon_{\mathrm{cr}}=\frac{1}{V_{f\_\mathrm{axial}}E_{f}}\left(\sigma -k\sigma_{\mathrm{to}}\right)-4\frac{\tau_{i}}{E_{f}}\frac{z^{2}}{r_{f}L}+\frac{4\tau_{i}}{E_{f}}\frac{{\left(y-2z\right)}^{2}}{r_{f}L}\\ \;+2\frac{\tau_{i}}{E_{f}}\frac{\left(L_{d}-2y+2z\right)\left(L_{d}+2y-2z-L\right)}{r_{f}L}-\left(\alpha_{c}-\alpha_{f}\right)\Delta Τ \end{array}$$

where *k* denotes the proportion of transverse yarns in the entire composite; and *σ*_to_ denotes the axial stress in the transverse yarns.

The unloading strain *ε*_cu_ and reloading strain *ε*_cr_ corresponding to interface slip Case 3 and Case 4 are determined by Equations (7) and (8), respectively [23].

(7) $$\varepsilon_{\mathrm{cu}}=\frac{1}{V_{f\_\mathrm{axial}}E_{f}}\left(\sigma -k\sigma_{\mathrm{to}}\right)+4\frac{\tau_{i}}{E_{f}}\frac{y^{2}}{r_{f}L}-2\frac{\tau_{i}}{E_{f}}\frac{{\left(2y-L/2\right)}^{2}}{r_{f}L}-\left(\alpha_{c}-\alpha_{f}\right)\Delta Τ$$

(8) $$\begin{array}{l} \varepsilon_{\mathrm{cr}}=\frac{1}{V_{f\_\mathrm{axial}}E_{f}}\left(\sigma -k\sigma_{\mathrm{to}}\right)-4\frac{\tau_{i}}{E_{f}}\frac{z^{2}}{r_{f}L}+4\frac{\tau_{i}}{E_{f}}\frac{{\left(y-2z\right)}^{2}}{r_{f}L}\\ \;-2\frac{\tau_{i}}{E_{f}}\frac{{\left(L/2-2y+2z\right)}^{2}}{r_{f}L}-\left(\alpha_{c}-\alpha_{f}\right)\Delta Τ \end{array}$$

### 2.3. Hysteresis Dissipated Energy

The fatigue hysteresis dissipated energy corresponding to different cycle number is determined by Equation (9) [23].

(9) $$U=\int_{\sigma_{\min}}^{\sigma_{\max}}\left[\varepsilon_{\mathrm{cu}}\left(\sigma \right)-\varepsilon_{\mathrm{cr}}\left(\sigma \right)\right]d\sigma$$

where *ε*_cu_ and *ε*_cr_ denote the unloading and reloading strain, respectively. Substituting the unloading and reloading strains of Equations (1)–(4) into (9), the fatigue hysteresis loss energy *U*_3_ of matrix cracking mode 3 can be obtained for different interface slips cases; substituting the unloading and reloading strains of Equations (5)–(8) into (9), the fatigue hysteresis loss energy *U*_5_ of matrix cracking mode 5 can also be obtained for different interface slip cases. The composite fatigue hysteresis loss energy *U*_c_ is determined by Equation (10) [23].

(10) $$U_{c}=\eta U_{3}+\left(1-\eta \right)U_{5}$$

where *η* denotes the composite damage parameter, i.e., the proportion of matrix cracking mode 3 in the entire matrix cracking modes.

By comparing experimental fatigue hysteresis dissipated energy with theoretical computational values, the interface shear stress of CMCs can be obtained [21]. The degradation rate *ψ* of interface shear stress can be determined by the Equation (11).

(11) $$\psi =\frac{\tau_{i}\left(N_{\mathrm{initial}}\right)-\tau_{i}\left(N_{\mathrm{final}}\right)}{N_{\mathrm{final}}-N_{\mathrm{initial}}}$$

where *N*_initial_ and *N*_final_ denote the initial and final cycle number for estimating interface shear stress, respectively; and *τ*_i_(*N*_initial_) and *τ*_i_(*N*_final_) denote the estimated interface shear stress at the initial and final cycle number, respectively.

## 3. Discussion

Under cyclic fatigue loading, the material properties, i.e., fiber volume content, peak stress, and damage state, i.e., matrix cracking mode proportion, affect the shape, location and area of hysteresis loops. The effect of these factors on interface slip and fatigue hysteresis dissipated energy evolution of matrix cracking mode 3 and mode 5, and composite would be analyzed.

### 3.1. Effect of Fiber Volume Fraction

**Figure 3:**
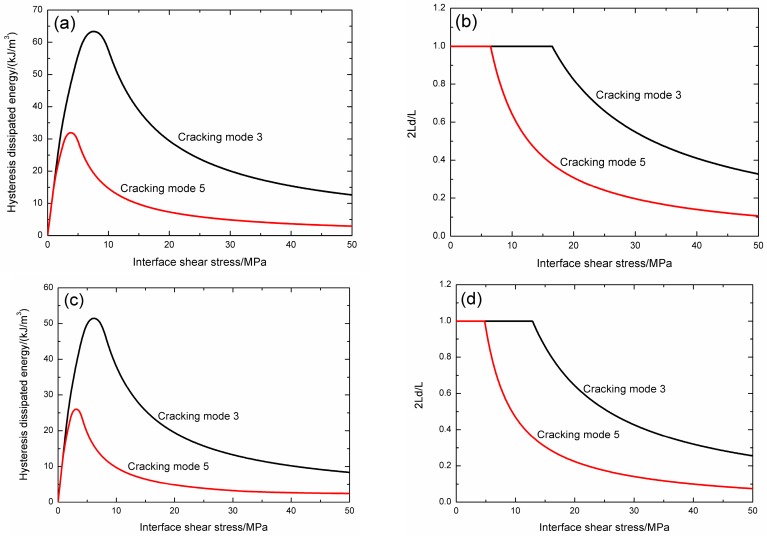

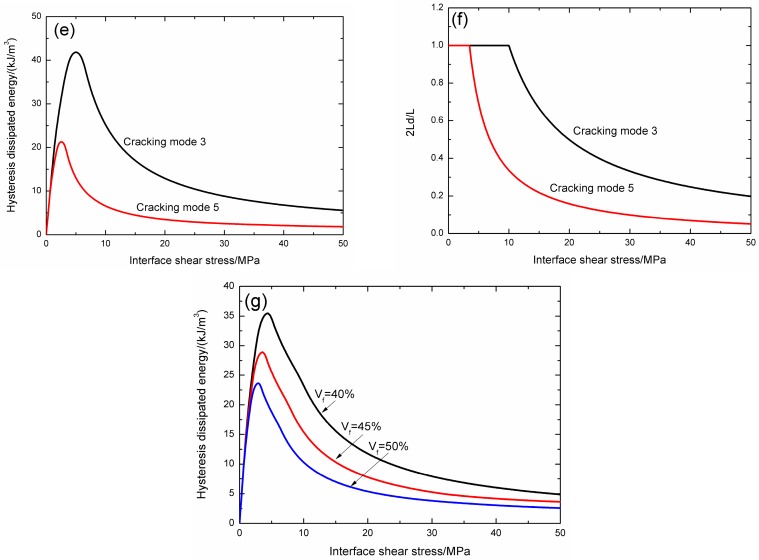
(**a**) The hysteresis dissipated energy versus interface shear stress curves of matrix cracking mode 3 and mode 5 when *V*_f_ = 40%; (**b**) the interface debonded length 2*L*_d_/*L* versus interface shear stress curves of matrix cracking mode 3 and mode 5 when *V*_f_ = 40%; (**c**) the hysteresis dissipated energy versus interface shear stress curves of matrix cracking mode 3 and mode 5 when *V*_f_ = 45%; (**d**) the interface debonded length 2*L*_d_/*L* versus interface shear stress curves of matrix cracking mode 3 and mode 5 when *V*_f_ = 45%; (**e**) the hysteresis dissipated energy versus interface shear stress curves of matrix cracking mode 3 and mode 5 when *V*_f_ = 50%; (**f**) the interface debonded length 2*L*_d_/*L* versus interface shear stress curves of matrix cracking mode 3 and mode 5 when *V*_f_ = 50%; and (**g**) the composite hysteresis dissipated energy versus interface shear stress curve when *V*_f_ = 40%, 45% and 50%, and *η* = 0.2.

The effect of fiber volume content on fatigue hysteresis dissipated energy evolution and interface slip of matrix cracking mode 3, mode 5 and the composite is illustrated in Figure 3.

When the fiber volume fraction is 40%, the fatigue hysteresis dissipated energy and interface debonded length 2*L*_d_/*L* versus interface shear stress curves of matrix cracking mode 3 and mode 5 are illustrated in Figure 3a,b. For matrix cracking mode 3, the hysteresis dissipated energy increases with decreasing interface shear stress from 12.6 kJ/m^3^ at the interface shear stress of 50 MPa to the peak value of 63.3 kJ/m^3^ at the interface shear stress of 7.6 MPa, and decreases to 0 kJ/m^3^ at the interface shear stress of 0 MPa, as shown in Figure 3a; and the interface debonded length 2*L*_d_/*L* increases with decreasing interface shear stress from 0.32 at the interface shear stress of 50 MPa to the peak value of 1.0 at the interface shear stress of 16.4 MPa, and remains to be constant of 1.0 until the interface shear stress of 0 MPa, as shown in Figure 3b. For matrix cracking mode 5, the hysteresis dissipated energy increases with decreasing interface shear stress from 2.9 kJ/m^3^ at the interface shear stress of 50 MPa to the peak value of 31.9 kJ/m^3^ at the interface shear stress of 3.8 MPa, and decreases to 0 kJ/m^3^ at the interface shear stress of 0 MPa, as shown in Figure 3a; and the interface debonded length 2*L*_d_/*L* increases with decreasing interface shear stress from 0.10 at the interface shear stress of 50 MPa to the peak value of 1.0 at the interface shear stress of 6.5 MPa, and remains to be constant of 1.0 until the interface shear stress of 0 MPa, as shown in Figure 3b.

When the fiber volume fraction is 45%, the fatigue hysteresis dissipated energy and interface debonded length 2*L*_d_/*L* versus interface shear stress curves of matrix cracking mode 3 and mode 5 are illustrated in Figure 3c,d. For matrix cracking mode 3, the hysteresis dissipated energy increases with decreasing interface shear stress from 8.3 kJ/m^3^ at the interface shear stress of 50 MPa to the peak value of 51.4 kJ/m^3^ at the interface shear stress of 6.1 MPa, and decreases to 0 kJ/m^3^ at the interface shear stress of 0 MPa, as shown in Figure 3c; and the interface debonded length 2*L*_d_/*L* increases with decreasing interface shear stress from 0.25 at the interface shear stress of 50 MPa to the peak value of 1.0 at the interface shear stress of 12.8 MPa, and remains to be constant of 1.0 until the interface shear stress of 0 MPa, as shown in Figure 3d. For matrix cracking mode 5, the hysteresis dissipated energy increases with decreasing interface shear stress from 2.4 kJ/m^3^ at the interface shear stress of 50 MPa to the peak value of 26 kJ/m^3^ at the interface shear stress of 3.1 MPa, and decreases to 0 kJ/m^3^ at the interface shear stress of 0 MPa, as shown in Figure 3c; and the interface debonded length 2*L*_d_/*L* increases with decreasing interface shear stress from 0.07 at the interface shear stress of 50 MPa to the peak value of 1.0 at the interface shear stress of 4.8 MPa, and remains to be constant of 1.0 until the interface shear stress of 0 MPa, as shown in Figure 3d.

When the fiber volume fraction is 50%, the fatigue hysteresis dissipated energy and interface debonded length 2*L*_d_/*L* versus interface shear stress curves of matrix cracking mode 3 and mode 5 are illustrated in Figure 3e,f. For matrix cracking mode 3, the hysteresis dissipated energy increases with decreasing interface shear stress from 5.5 kJ/m^3^ at the interface shear stress of 50 MPa to the peak value of 41.8 kJ/m^3^ at the interface shear stress of 5 MPa, and decreases to 0 kJ/m^3^ at the interface shear stress of 0 MPa, as shown in Figure 3e; and the interface debonded length 2*L*_d_/*L* increases with decreasing interface shear stress from 0.19 at the interface shear stress of 50 MPa to the peak value of 1.0 at the interface shear stress of 9.9 MPa, and remains to be constant of 1.0 until the interface shear stress of 0 MPa, as shown in Figure 3f. For matrix cracking mode 5, the hysteresis dissipated energy increases with decreasing interface shear stress from 1.8 kJ/m^3^ at the interface shear stress of 50 MPa to the peak value of 21.2 kJ/m^3^ at the interface shear stress of 2.5 MPa, and decreases to 0 kJ/m^3^ at the interface shear stress of 0 MPa, as shown in Figure 3e; and the interface debonded length 2*L*_d_/*L* increases with decreasing interface shear stress from 0.05 at the interface shear stress of 50 MPa to the peak value of 1.0 at the interface shear stress of 3.4 MPa, and remains to be constant of 1.0 until the interface shear stress of 0 MPa, as shown in Figure 3f.

When matrix cracking mode 3 proportion *η* is 0.2, the composite fatigue hysteresis dissipated energy versus interface shear stress curves when the fiber volume fraction is 40%, 45% and 50% are illustrated in Figure 3g. When the fiber volume fraction is 40%, the composite hysteresis dissipated energy increases with decreasing interface shear stress from 4.8 kJ/m^3^ at the interface shear stress of 50 MPa to the peak value of 35.4 kJ/m^3^ at the interface shear stress of 4.4 MPa, and decreases to 0 kJ/m^3^ at the interface shear stress of 0 MPa; when the fiber volume fraction is 45%, the composite hysteresis dissipated energy increases with decreasing interface shear stress from 3.6 kJ/m^3^ at the interface shear stress of 50 MPa to the peak value of 28.8 kJ/m^3^ at the interface shear stress of 3.6 MPa, and decreases to 0 kJ/m^3^ at the interface shear stress of 0 MPa; and when the fiber volume fraction is 50%, the composite hysteresis dissipated energy increases with decreasing interface shear stress from 2.5 kJ/m^3^ at the interface shear stress of 50 MPa to the peak value of 23.6 kJ/m^3^ at the interface shear stress of 2.9 MPa, and decreases to 0 kJ/m^3^ at the interface shear stress of 0 MPa.

When fiber volume content increases, the hysteresis dissipated energy of matrix cracking mode 3, mode 5 and the composite at the same interface shear stress decrease, due to less interface debonding and frictional slip between fibers and the matrix.

### 3.2. Effect of Fatigue Peak Stress

**Figure 4:**
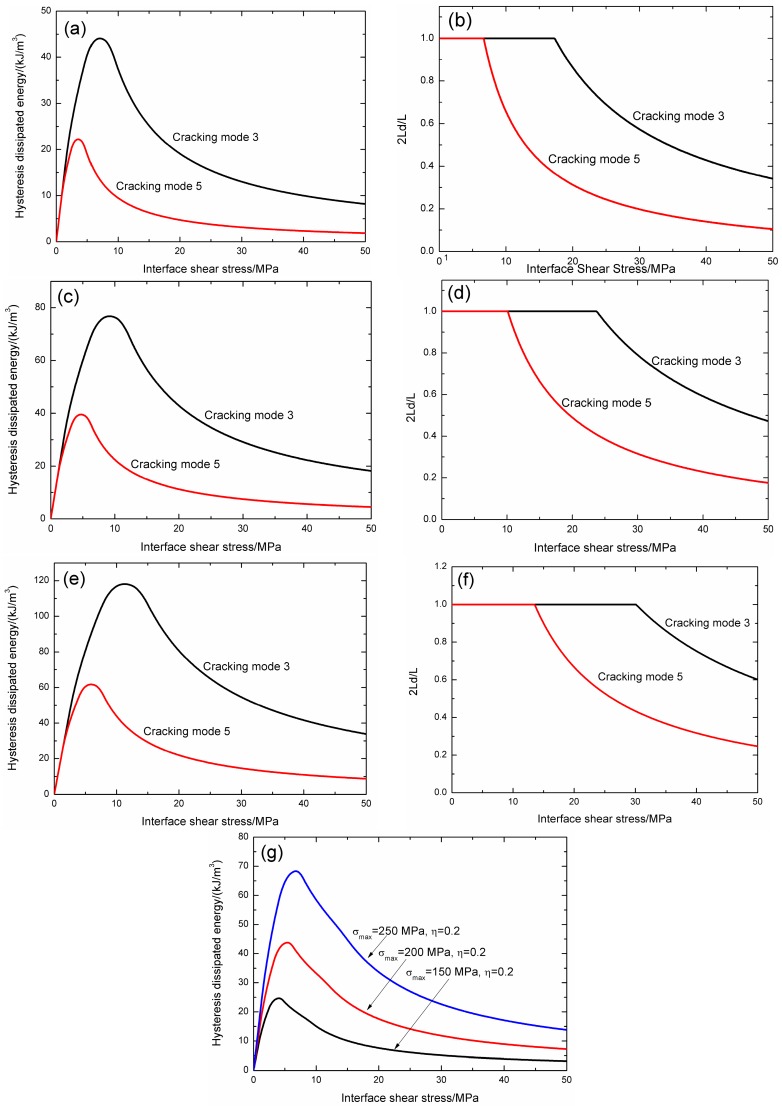
(**a**) The hysteresis dissipated energy versus interface shear stress curves of matrix cracking mode 3 and mode 5 when *σ*_max_ = 150 MPa; (**b**) the interface debonded length 2*L*_d_/*L* versus interface shear stress curves of matrix cracking mode 3 and mode 5 when *σ*_max_ = 150 MPa; (**c**) the hysteresis dissipated energy versus interface shear stress curves of matrix cracking mode 3 and mode 5 when *σ*_max_ = 200 MPa; (**d**) the interface debonded length 2*L*_d_/*L* versus interface shear stress curves of matrix cracking mode 3 and mode 5 when *σ*_max_ = 200 MPa; (**e**) the hysteresis dissipated energy versus interface shear stress curves of matrix cracking mode 3 and mode 5 when *σ*_max_ = 250 MPa; (**f**) the interface debonded length 2*L*_d_/*L* versus interface shear stress curves of matrix cracking mode 3 and mode 5 when *σ*_max_ = 250 MPa; and (**g**) the composite hysteresis dissipated energy versus interface shear stress curves when *σ*_max_ = 150, 200 and 250 MPa, and *η* = 0.2.

The effect of peak stress on fatigue hysteresis dissipated energy evolution and interface slip of matrix cracking mode 3, mode 5 and composite is illustrated in Figure 4.

When the fatigue peak stress is 150 MPa, the fatigue hysteresis dissipated energy and interface debonded length 2*L*_d_/*L* versus interface shear stress curves of matrix cracking mode 3 and mode 5 are illustrated in Figure 4a,b. For matrix cracking mode 3, the hysteresis dissipated energy increases with decreasing interface shear stress from 8.2 kJ/m^3^ at the interface shear stress of 50 MPa to the peak value of 44 kJ/m^3^ at the interface shear stress of 7 MPa, and decreases to 0 kJ/m^3^ at the interface shear stress of 0 MPa, as shown in Figure 4a; and the interface debonded length 2*L*_d_/*L* increases with decreasing interface shear stress from 0.34 at the interface shear stress of 50 MPa to the peak value of 1.0 at the interface shear stress of 17.2 MPa, and remains to be constant of 1.0 until the interface shear stress of 0 MPa, as shown in Figure 4b. For matrix cracking mode 5, the hysteresis dissipated energy increases with decreasing interface shear stress from 1.9 kJ/m^3^ at the interface shear stress of 50 MPa to the peak value of 22.2 kJ/m^3^ at the interface shear stress of 3.5 MPa, and decreases to 0 kJ/m^3^ at the interface shear stress of 0 MPa, as shown in Figure 4a; and the interface debonded length 2*L*_d_/*L* increases with decreasing interface shear stress from 0.1 at the interface shear stress of 50 MPa to the peak value of 1.0 at the interface shear stress of 6.6 MPa, and remains to be constant of 1.0 until the interface shear stress of 0 MPa, as shown in Figure 4b.

When *σ*_max_ = 200 MPa, the fatigue hysteresis dissipated energy and interface debonded length 2*L*_d_/*L* versus interface shear stress curves of matrix cracking mode 3 and mode 5 are illustrated in Figure 4c,d. For matrix cracking mode 3, the hysteresis dissipated energy increases with decreasing interface shear stress from 18.2 kJ/m^3^ at the interface shear stress of 50 MPa to the peak value of 76.7 kJ/m^3^ at the interface shear stress of 9.2 MPa, and decreases to 0 kJ/m^3^ at the interface shear stress of 0 MPa, as shown in Figure 4c; and the interface debonded length 2*L*_d_/*L* increases with decreasing interface shear stress from 0.47 at the interface shear stress of 50 MPa to the peak value of 1.0 at the interface shear stress of 23.7 MPa, and remains to be constant of 1.0 until the interface shear stress of 0 MPa, as shown in Figure 4d. For matrix cracking mode 5, the hysteresis dissipated energy increases with decreasing interface shear stress from 4.5 kJ/m^3^ at the interface shear stress of 50 MPa to the peak value of 39.5 kJ/m^3^ at the interface shear stress of 4.7 MPa, and decreases to 0 kJ/m^3^ at the interface shear stress of 0 MPa, as shown in Figure 4c; and the interface debonded length 2*L*_d_/*L* increases with decreasing interface shear stress from 0.17 at the interface shear stress of 50 MPa to the peak value of 1.0 at the interface shear stress of 10 MPa, and remains to be constant of 1.0 until the interface shear stress of 0 MPa, as shown in Figure 4d.

When *σ*_max_ = 250 MPa, the fatigue hysteresis dissipated energy and interface debonded length 2*L*_d_/*L* versus interface shear stress curves of matrix cracking mode 3 and mode 5 are illustrated in Figure 4e,f. For matrix cracking mode 3, the hysteresis dissipated energy increases with decreasing interface shear stress from 33.8 kJ/m^3^ at the interface shear stress of 50 MPa to the peak value of 118.1 kJ/m^3^ at the interface shear stress of 11.3 MPa, and decreases to 0 kJ/m^3^ at the interface shear stress of 0 MPa, as shown in Figure 4e; and the interface debonded length 2*L*_d_/*L* increases with decreasing interface shear stress from 0.6 at the interface shear stress of 50 MPa to the peak value of 1.0 at the interface shear stress of 30.1 MPa, and remains to be constant of 1.0 until the interface shear stress of 0 MPa, as shown in Figure 4f. For matrix cracking mode 5, the hysteresis dissipated energy increases with decreasing interface shear stress from 8.7 kJ/m^3^ at the interface shear stress of 50 MPa to the peak value of 61.7 kJ/m^3^ at the interface shear stress of 5.9 MPa, and decreases to 0 kJ/m^3^ at the interface shear stress of 0 MPa, as shown in Figure 4e; and the interface debonded length 2*L*_d_/*L* increases with decreasing interface shear stress from 0.24 at the interface shear stress of 50 MPa to the peak value of 1.0 at the interface shear stress of 13.5 MPa, and remains to be constant of 1.0 until the interface shear stress of 0 MPa, as shown in Figure 4f.

When the proportion of matrix cracking mode 3 is *η* = 0.2, the composite hysteresis dissipated energy versus interface shear stress curves when the fatigue peak stress is 150 and 200 MPa are illustrated in Figure 4g. When the fatigue peak stress is 150 MPa, the composite hysteresis dissipated energy increases with decreasing interface shear stress from 3.1 kJ/m^3^ at the interface shear stress of 50 MPa to the peak value of 24.6 kJ/m^3^ at the interface shear stress of 4.1 MPa, and decreases to 0 kJ/m^3^ at the interface shear stress of 0 MPa; when the fatigue peak stress is 200 MPa, the composite hysteresis dissipated energy increases with decreasing interface shear stress from 7.2 kJ/m^3^ at the interface shear stress of 50 MPa to the peak value of 43.7 kJ/m^3^ at the interface shear stress of 5.4 MPa, and decreases to 0 kJ/m^3^ at the interface shear stress of 0 MPa; and when the fatigue peak stress is 250 MPa, the composite hysteresis dissipated energy increases with decreasing interface shear stress from 13.8 kJ/m^3^ at the interface shear stress of 50 MPa to the peak value of 68.3 kJ/m^3^ at the interface shear stress of 6.8 MPa, and decreases to 0 kJ/m^3^ at the interface shear stress of 0 MPa.

When the fatigue peak stress increases, the hysteresis dissipated energy of matrix cracking mode 3, mode 5 and composite at the same interface shear stress increase, due to more interface debonding and frictional slip between fibers and the matrix.

### 3.3. Effect of Matrix Cracking Mode Proportion

**Figure 5:**
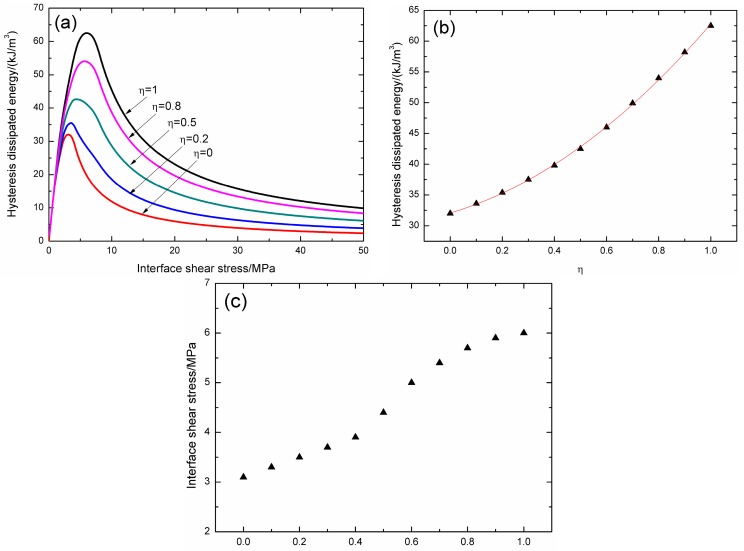
Effect matrix cracking mode proportion, i.e., *η* = 0, 0.2, 0.5, 0.8 and 1.0, on (**a**) the hysteresis dissipated energy versus interface shear stress curve; (**b**) the peak value of composite hysteresis dissipated energy versus *η* curve; and (**c**) the corresponding interface shear stress versus *η* curve.

The effect of matrix cracking proportion *η* on fatigue hysteresis dissipated energy versus interface shear stress curves is illustrated in Figure 5a. When *η* is 0, there is only matrix cracking mode 5 in the composite, the composite hysteresis dissipated energy increases from 2.3 kJ/m^3^ at the interface shear stress of 50 MPa to the peak value of 32 kJ/m^3^ at the interface shear stress of 3.1 MPa, as shown in Figure 5a; when *η* is 1, there is only matrix cracking mode 3 in the composite, the composite hysteresis dissipated energy increases from 9.8 kJ/m^3^ at the interface shear stress of 50 MPa to the peak value of 62.5 kJ/m^3^ at the interface shear stress of 6 MPa, as shown in Figure 5a; and when 0 < *η* < 1, there are both matrix cracking mode 3 and mode 5 in the composite, when *η* is 0.2, the composite hysteresis dissipated energy increases from 3.8 kJ/m^3^ at the interface shear stress of 50 MPa to the peak value of 35.4 kJ/m^3^ at the interface shear stress of 3.5 MPa; when *η* is 0.5, the composite hysteresis dissipated energy increases from 6.1 kJ/m^3^ at the interface shear stress of 50 MPa to the peak value of 42.5 kJ/m^3^ at the interface shear stress of 4.4 MPa; and when *η* is 0.8, the composite hysteresis dissipated energy increases from 8.3 kJ/m^3^ at the interface shear stress of 50 MPa to the peak value of 54 kJ/m^3^ at the interface shear stress of 5.7 MPa, as shown in Figure 5a. With increasing of matrix cracking mode 3 proportion *η*, the peak value of hysteresis dissipated energy increases, as shown in Figure 5b; and the interface shear stress corresponding to the peak value of hysteresis dissipated energy also increases, as shown in Figure 5c.

When matrix cracking mode 3 proportion *η* increases, the composite hysteresis dissipated energy and the corresponding interface shear stress increase, which indicates that the hysteresis dissipated energy can approach to the peak value at high interface shear stress.

## 4. Experimental Comparisons

### 4.1. 2D C/SiC Composite

#### 4.1.1. Room Temperature

Li [21] investigated the tension–tension fatigue behavior of cross-ply C/SiC composite at room temperature. The T–700™ carbon (Toray Institute Inc., Tokyo, Japan) fiber-reinforced silicon carbide matrix composites (C/SiC CMCs) were manufactured by the hot-pressing method. The dog bone-shaped specimens were cut from 150 mm × 150 mm panels by water jet cutting. The test specimens were further coated with SiC of about 20 μm thick by chemical vapor deposition (CVD) to prevent oxidation at elevated temperatures. These processing steps resulted in a material having bulk density about 2.0 g/cm^3^, and an open porosity less than 5%. The fatigue tests were conducted in force control with a stress ratio (i.e., minimum stress/maximum stress) of 0.1, and a sinusoidal waveform loading frequency of 10 Hz. The material properties are given by: *V*_f_ = 40%, *E*_f_ = 230 GPa, *E*_m_ = 350 GPa, *r*_f_ = 3.5 μm, *α*_f_ = −0.38 × 10^−6^/°C, *α*_m_ = 2.8 × 10^−6^/°C, and Δ*T* = −1000 °C.

**Figure 6:**
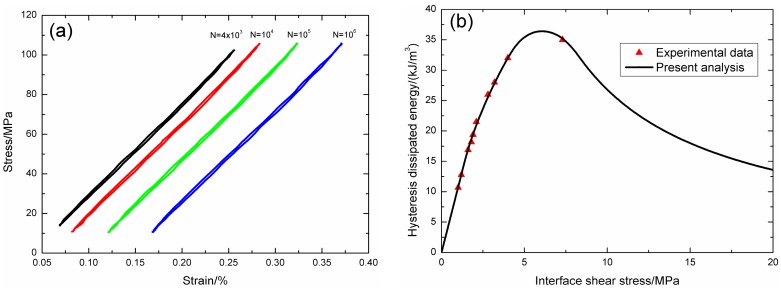
(**a**) The experimental fatigue hysteresis loops corresponding to different cycle number; and (**b**) the experimental and theoretical fatigue hysteresis dissipated energy versus interface shear stress curve of cross-ply C/SiC composite under *σ*_max_ = 105 MPa at room temperature.

Under fatigue peak stress of *σ*_max_ = 105 MPa, the fatigue hysteresis loops corresponding to different applied cycles are illustrated in Figure 6a, in which the proportion of matrix cracking mode 3 is *η* = 0.8. The experimental and theoretical fatigue hysteresis dissipated energy as a function of interface shear stress is shown in Figure 6b. The theoretical fatigue hysteresis dissipated energy increases with decreasing interface shear stress to the peak value of 36.4 kJ/m^3^, and decreases with decreasing interface shear stress to 0 kJ/m^3^. The experimental fatigue hysteresis dissipated energy of the 1st cycle lies in the right part of the fatigue hysteresis dissipated energy versus interface shear stress curve. The fatigue hysteresis loop of the 1st cycle corresponds to interface slip Case 2, i.e., interface partially debonds and fiber slides partially relative to matrix in the interface debonded region. With the number of applied cycles increasing, interface shear stress decreases with increasing cycle number due to interface wear. Staehler et al. [7] observed the interface wear as a result of sliding contact between individual 0° fibers within a tow at the loading frequency of 4 Hz. However, no fiber surface abrasions were evident in specimens failed under monotonic tensile loading conditions. Upon cyclic loading, when matrix cracks are present, the matrix slides past the intact fibers. These sliding displacements change the interface shear stress and cause further debonding. The reductions in interface shear stress are attributed to interface wear operating in the fiber coating, especially at those contacts subject to high pressure. The wear process is facilitated by the temperature rise that occurs along the interface, as frictional dissipation proceeds. At high frequencies, the increase in temperature can be large enough to oxidize the fiber coating at room temperature [7]. The wear debris produced during the wear process are particles which have a mild lubricating effect and are easily broken down further during fatigue loading cycles. By comparing experimental fatigue hysteresis dissipated energy with theoretical values, the interface shear stress corresponding to different applied cycles can be estimated, as shown in Table 1. The fatigue hysteresis loop of the 100th cycle corresponds to interface slip Case 4, i.e., interface completely debonds and fiber slides completely relative to matrix in the interface debonded region.

Shuler et al. [6] investigated the tension–tension fatigue behavior of 2D woven C/SiC composite at room temperature. The T–300™ carbon (Toray Institute Inc., Tokyo, Japan) fiber-reinforced silicon carbide matrix composites (C/SiC CMCs) were processed by chemical vapor infiltration (CVI) of SiC into woven 0°/90° carbon fiber preforms. The test specimens were cut from 200 mm × 200 mm composite panels using diamond tooling into a dog-bone configuration. The composite density ranges from 1.93 to 1.98 g/cm^3^. The fatigue tests were performed under force control at a sinusoidal loading frequency of 10 Hz, with a stress ratio (i.e., minimum stress/maximum stress) of 0.1. The material properties are given by: *V*_f_ = 45%, *E*_f_ = 230 GPa, *E*_m_ = 350 GPa, *r*_f_ = 3.5 μm, *α*_f_ = 0, *α*_m_ = 4.6 × 10^−6^/K, and Δ*T* = −1000 K.

**Figure 7:**
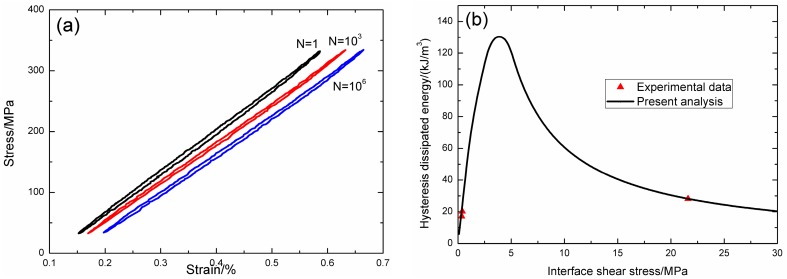
(**a**) The experimental fatigue hysteresis loops corresponding to different cycle number; and (**b**) the experimental and theoretical fatigue hysteresis dissipated energy versus interface shear stress curve of 2D woven C/SiC composite under *σ*_max_ = 335 MPa at room temperature.

Under fatigue peak stress of *σ*_max_ = 335 MPa, the fatigue hysteresis loops corresponding to different applied cycles are illustrated in Figure 7a, in which the proportion of matrix cracking mode 3 is *η* = 0.2. The experimental and theoretical fatigue hysteresis dissipated energy as a function of interface shear stress is shown in Figure 7b. The theoretical fatigue hysteresis dissipated energy increases with decreasing interface shear stress to the peak value of 130.4 kJ/m^3^, and decreases with decreasing interface shear stress to 0 kJ/m^3^. The experimental fatigue hysteresis dissipated energy of the 1st cycle lies in the right part of the fatigue hysteresis dissipated energy versus interface shear stress curve. The fatigue hysteresis loop of the 1st cycle corresponds to interface slip Case 2, i.e., interface partially debonds, and fiber slides partially relative to matrix in the interface debonded region. With the number of applied cycles increasing, interface shear stress decreases with increasing cycle number due to interface wear. By comparing experimental fatigue hysteresis dissipated energy with theoretical values, the interface shear stress corresponding to different applied cycles can be estimated, as shown in Table 2. The fatigue hysteresis loops of the 1000th and 1,000,000th applied cycle correspond to interface slip Case 4, i.e., interface completely debonds and fiber slides completely relative to matrix in the interface debonded region.

Staehler et al. [7] investigated the tension–tension fatigue behavior of 0°/90° plain-weave C/SiC composite at room temperature. The T–300™ carbon (Toray Institute Inc., Tokyo, Japan) fiber-reinforced silicon carbide matrix composites (C/SiC CMCs) were manufactured using the CVI process. The 0°/90° fiber preform was given a pyrolytic carbon coating to promote composite toughness. The composite bulk density was about 2.0 g/cm^3^. After machining, each test specimen received a SiC seal coat via CVD. The fatigue loading was in a sinusoidal waveform with a loading frequency of 40 Hz. The fatigue stress ratio was 0.05. The material properties of 2D woven C/SiC composite are given by: *V*_f_ = 45%, *E*_f_ = 225 GPa, *E*_m_ = 430 GPa, *r*_f_ = 3.5 μm, *α*_f_ = 1.0 × 10^−6^/°C, *α*_m_ = 4.8 × 10^−6^/°C, and Δ*T* = −1000 °C.

**Figure 8:**
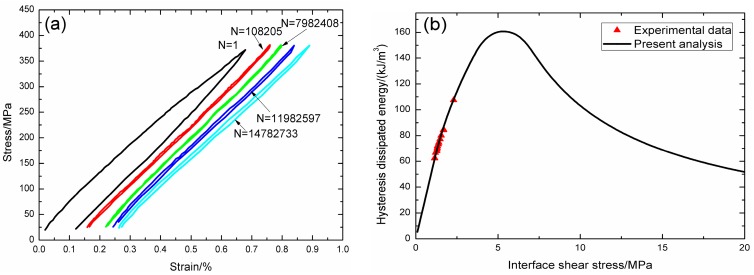
(**a**) The experimental fatigue hysteresis loops corresponding to different cycle number; and (**b**) the experimental and theoretical fatigue hysteresis dissipated energy versus interface shear stress curve of 2D woven C/SiC composite under *σ*_max_ = 375 MPa at room temperature.

Under fatigue peak stress of *σ*_max_ = 375 MPa, the fatigue hysteresis loops corresponding to different applied cycles are illustrated in Figure 8a, in which the proportion of matrix cracking mode 3 is *η* = 0.2. The experimental and theoretical fatigue hysteresis dissipated energy as a function of interface shear stress is shown in Figure 8b. The theoretical fatigue hysteresis dissipated energy increases with decreasing interface shear stress to the peak value of 160.7 kJ/m^3^, and then decreases with decreasing interface shear stress to 0 kJ/m^3^. The experimental fatigue hysteresis dissipated energy of the 2nd cycle lies in the left part of the fatigue hysteresis dissipated energy versus interface shear stress curve. The fatigue hysteresis loop of the 2nd cycle corresponds to interface slip Case 4, i.e., the interface completely debonds, and fiber slips completely relative to matrix in the interface debonded region. By comparing experimental fatigue hysteresis dissipated energy with theoretical values, the interface shear stress corresponding to different applied cycles can be estimated, as shown in Table 3.

Li [5] investigated the tension–tension fatigue behavior of 2D woven C/SiC composite at room temperature. The HTA carbon (Toho Tenax, Tokyo, Japan) fiber-reinforced silicon carbide matrix composites (C/SiC CMCs) were fabricated by laminating 2D plain weave carbon fabrics by liquid silicon infiltration (LSI) method. The fatigue loading was in a sinusoidal waveform with a loading frequency of 10 Hz, and the fatigue stress ratio was 0.1. The material properties of 2D woven C/SiC composite are given by: *V*_f_ = 50%, *E*_f_ = 230 GPa, *E*_m_ = 350 GPa, *r*_f_ = 3.5 μm, *α*_f_ = −0.38 × 10^−6^/°C, *α*_m_ = 2.8 × 10^−6^/°C, and Δ*T* = −1000 °C.

**Figure 9:**
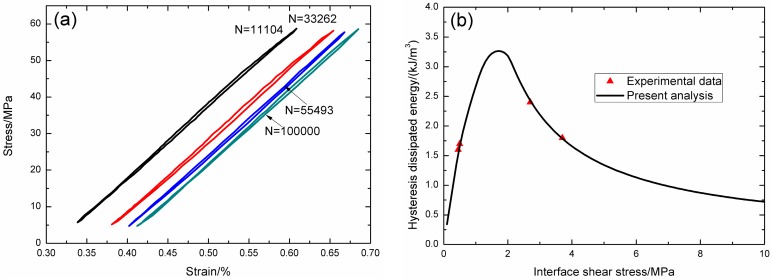
(**a**) The experimental fatigue hysteresis loops corresponding to different cycle number; and (**b**) the experimental and theoretical fatigue hysteresis dissipated energy versus interface shear stress curve of 2D woven C/SiC composite under *σ*_max_ = 57 MPa at room temperature.

Under fatigue peak stress of *σ*_max_ = 57 MPa, the fatigue hysteresis loops corresponding different applied cycles are illustrated in Figure 9a, in which the proportion of matrix cracking mode 3 is *η* = 0.2. The experimental and theoretical fatigue hysteresis dissipated energy as a function of interface shear stress is shown in Figure 9b. The theoretical fatigue hysteresis dissipated energy increases with decreasing interface shear stress to the peak value of 3.2 kJ/m^3^, and then decreases with decreasing interface shear stress to 0 kJ/m^3^. The experimental fatigue hysteresis dissipated energy of the 11,104th and 33,262nd applied cycles lies in the right part of the fatigue hysteresis dissipated energy versus interface shear stress curve. The fatigue hysteresis loop of the 1st cycle corresponds to interface slip Case 2, i.e., the interface partially debonds, and fiber slips partially relative to matrix in the interface debonded region. With the number of applied cycles increasing, interface shear stress decreases with increasing cycle number due to interface wear. By comparing experimental fatigue hysteresis dissipated energy with theoretical values, the interface shear stress corresponding to different applied cycles can be estimated, as shown in Table 4. The fatigue hysteresis loops of the 55,493rd and 100,000th applied cycles correspond to interface slip Case 4, i.e., the interface completely debonds, and fiber slips completely relative to matrix in the interface debonded region.

#### 4.1.2. Elevated Temperature

Li [21] investigated the tension–tension fatigue behavior of cross-ply C/SiC composite at 800 °C in air. The fatigue loading was in a sinusoidal waveform and a loading frequency of 10 Hz, and the fatigue stress ratio was 0.1. The material properties of cross-ply C/SiC composite are given by: *V*_f_ = 40%, *E*_f_ = 230 GPa, *E*_m_ = 350 GPa, *r*_f_ = 3.5 μm, *α*_f_ = −0.38 × 10^−6^/°C, *α*_m_ = 2.8 × 10^−6^/°C, and Δ*T* = −200 °C.

**Figure 10:**
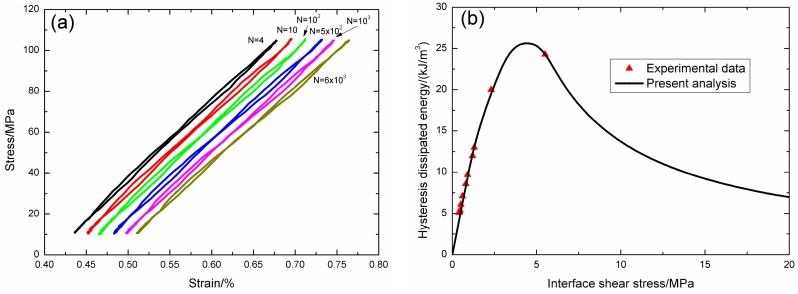
(**a**) The experimental fatigue hysteresis loops corresponding to different cycle number; and (**b**) the experimental and theoretical fatigue hysteresis dissipated energy versus interface shear stress curve of cross-ply C/SiC composite under *σ*_max_ = 105 MPa at 800 °C in air.

Under fatigue peak stress of *σ*_max_ = 105 MPa, the fatigue hysteresis loops corresponding to different applied cycles are illustrated in Figure 10a, in which the proportion of matrix cracking mode 3 is *η* = 0.8. The experimental and theoretical fatigue hysteresis dissipated energy as a function of interface shear stress is shown in Figure 10b. The theoretical fatigue hysteresis dissipated energy increases with decreasing interface shear stress to the peak value of 25.6 kJ/m^3^, and then decreases with decreasing interface shear stress to 0 kJ/m^3^. The experimental fatigue hysteresis dissipated energy of the 1st cycle lies in the right part of the fatigue hysteresis dissipated energy versus interface shear stress curve. The fatigue hysteresis loop of the 1st cycle corresponds to interface slip Case 2, i.e., the interface partially debonds, and fiber slips partially relative to matrix in the interface debonded region. With the number of applied cycles increasing, interface shear stress decreases with increasing cycle number due to interface wear and interface oxidation. For interface oxidation, the oxidation of fiber coating is a process such as,

(12) C+O2→CO2 or C+12O2→CO

i.e., the oxidation removes material in the form of a gas.

By comparing experimental fatigue hysteresis dissipated energy with theoretical values, the interface shear stress corresponding to different applied cycles can be estimated, as shown in Table 5. The fatigue hysteresis loop of the 100th cycle corresponds to interface slip Case 4, i.e., the interface completely debonds, and fiber slips completely relative to matrix in the interface debonded region.

Mall and Engesser [8] investigated the tension–tension fatigue behavior of 0°/90° plain-weave C/SiC composite at 550 °C in air. The T–300™ carbon (Toray Institute Inc., Tokyo, Japan) fiber-reinforced silicon carbide matrix composites (C/SiC CMCs) were manufactured using the CVI method with the reinforcement from plain-weave cloth in a [0/90] lay-up. The T–300 carbon fiber preform was given a pyrolytic carbon coating to promote toughness. The composite density was to be 2.0 g/cm^3^. The test specimens were cut from 216 mm × 216 mm composite panels using diamond grinding into a dog-bone configuration. After the machining and cutting, the test specimens were then seal coated with SiC via CVD process. The fatigue tests were conducted in the force control with a stress ratio (i.e., minimum stress/maximum stress) of 0.05, and a sinusoidal waveform loading frequency of 0.1 Hz. The fatigue stress ratio was 0.1. The material properties of 2D woven C/SiC composite are given by: *V*_f_ = 45%, *E*_f_ = 225 GPa, *E*_m_ = 430 GPa, *r*_f_ = 3.5 μm, *α*_f_ = 1.0 × 10^−6^/°C, *α*_m_ = 4.8 × 10^−6^/°C, and Δ*T* = −500 °C.

**Figure 11:**
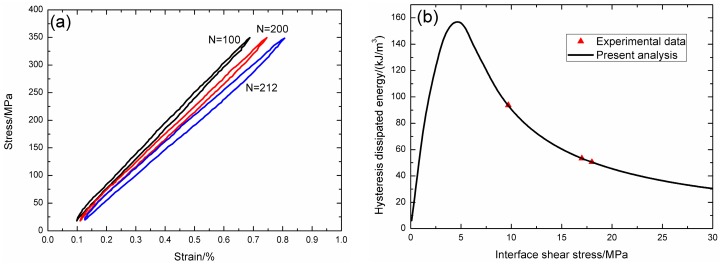
(**a**) The experimental fatigue hysteresis loops corresponding to different cycle number; and (**b**) the experimental and theoretical fatigue hysteresis dissipated energy versus interface shear stress curve of 2D woven C/SiC composite under *σ*_max_ = 350 MPa at 550 °C in air.

Under fatigue peak stress of *σ*_max_ = 350 MPa, the fatigue hysteresis loops corresponding to different applied cycles are illustrated in Figure 11a, in which the proportion of matrix cracking mode 3 is *η* = 0.2. The experimental and theoretical fatigue hysteresis dissipated energy as a function of interface shear stress is shown in Figure 11b. The theoretical fatigue hysteresis dissipated energy increases with decreasing interface shear stress to the peak value of 156.7 kJ/m^3^, and then decreases with decreasing interface shear stress to 0 kJ/m^3^. The experimental fatigue hysteresis dissipated energy of the 100th cycle lies in the right part of the fatigue hysteresis dissipated energy versus interface shear stress curve. The fatigue hysteresis loops of the 100th, 200th, and 212th applied cycles correspond to interface slip Case 2, i.e., the interface partially debonds, and fiber slips partially relative to matrix in the interface debonded region. With the number of applied cycles increasing, interface shear stress decreases with increasing cycle number due to interface wear and interface oxidation. By comparing experimental fatigue hysteresis dissipated energy with theoretical values, the interface shear stress corresponding to different applied cycles can be estimated, as shown in Table 6.

Rodrigues et al. [9] investigated the tension–tension fatigue behavior of 2D C/SiC composite at 1200 °C in vacuum. The T–300™ carbon (Toray Institute Inc., Tokyo, Japan) fiber-reinforced silicon carbide matrix composites (C/SiC CMCs) was a superposition of several plain weave layers of carbon fiber bundles embedded in a SiC matrix through a CVI process. The composite density is about 2.0 g/cm^3^. The fatigue tests were performed under force control with a triangular loading cycle with a loading frequency of 10 Hz, and a stress ratio (i.e., minimum stress/maximum stress) of 0.1. The material properties of 2D woven C/SiC composite are given by: *V*_f_ = 40%, *E*_f_ = 225 GPa, *E*_m_ = 350 GPa, *r*_f_ = 3.5 μm, *α*_f_ = 1.0 × 10^−6^/°C, *α*_m_ = 4.8 × 10^−6^/°C, and Δ*T* = −100 °C.

**Figure 12:**
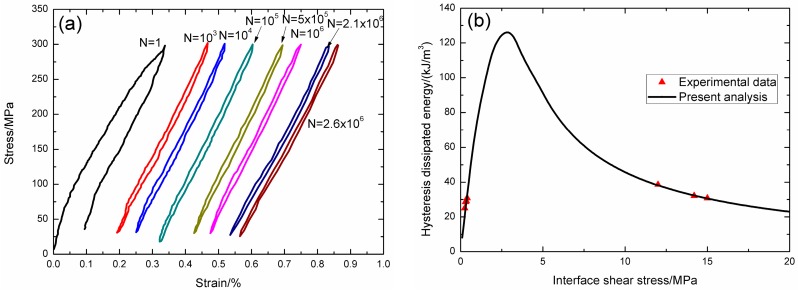
(**a**) The experimental fatigue hysteresis loops corresponding to different cycle number; and (**b**) the experimental and theoretical fatigue hysteresis dissipated energy versus interface shear stress curve of 2D woven C/SiC composite under *σ*_max_ = 300 MPa at 1200 °C in vacuum.

Under fatigue peak stress of *σ*_max_ = 300 MPa, the fatigue hysteresis loops corresponding to different applied cycles are illustrated in Figure 12a, in which the proportion of matrix cracking mode 3 is *η* = 0.2. The experimental and theoretical fatigue hysteresis dissipated energy as a function of interface shear stress is shown in Figure 12b. The theoretical fatigue hysteresis dissipated energy increases with decreasing interface shear stress to the peak value of 126 kJ/m^3^, and then decreases with decreasing interface shear stress to 0 kJ/m^3^. The experimental fatigue hysteresis dissipated energy of the 1000th, 100,000th, and 1,000,000th applied cycle lies in the right part of the fatigue hysteresis dissipated energy versus interface shear stress curve. The fatigue hysteresis loop of the 1st cycle corresponds to interface slip Case 2, i.e., the interface partially debonds, and fiber slips partially relative to matrix in the interface debonded region. With the number of applied cycles increasing, interface shear stress decreases with increasing cycle number due to interface wear and interface oxidation. By comparing experimental fatigue hysteresis dissipated energy with theoretical values, the interface shear stress corresponding to different applied cycles can be estimated, as shown in Table 7. The fatigue hysteresis loops of the 500,000th, 1,000,000th, 2,100,000th, and 2,600,000th applied cycles correspond to interface slip Case 4, i.e., the interface completely debonds, and fiber slips completely relative to matrix in the interface debonded region.

### 4.2. 2D SiC/SiC Composite

#### 4.2.1. Room Temperature

Shi [11] investigated the tension–tension fatigue behavior of 2D woven SiC/SiC composite at room temperature. The Hi-Nicalon™ SiC (Nippon Carbon Co., Ltd., Tokyo, Japan) fiber-reinforced silicon carbide matrix composites (SiC/SiC CMCs) were manufactured using CVI method whereby fiber fabrics are first CVD coated with BN before several cycles of CVI to deposit SiC matrix on the coated fiber fabrics. The fatigue loading was in a sinusoidal waveform and a loading frequency of 1.0 Hz, and the fatigue stress ratio was 0.1. The material properties of 2D woven SiC/SiC composite are given by: *V*_f_ = 40%, *E*_f_ = 270 GPa, *E*_m_ = 300 GPa, *r*_f_ = 7.0 μm, *α*_f_ = 5.1 × 10^−6^/°C, *α*_m_ = 4.7 × 10^−6^/°C, and Δ*T* = −1000 °C.

**Figure 13:**
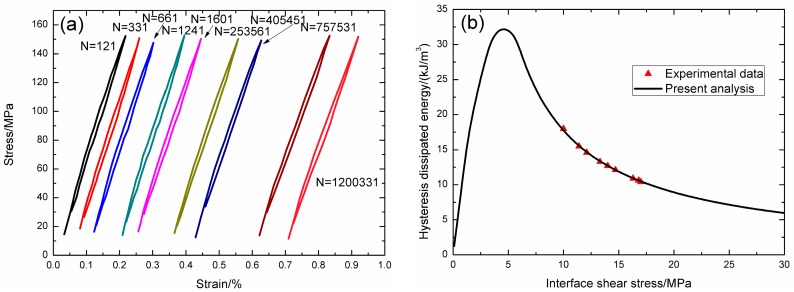
(**a**) The experimental fatigue hysteresis loops corresponding to different cycle number; and (**b**) the experimental and theoretical fatigue hysteresis dissipated energy versus interface shear stress curve of 2D woven SiC/SiC composite under *σ*_max_ = 150 MPa at room temperature.

Under fatigue peak stress of *σ*_max_ = 150 MPa, the fatigue hysteresis loops corresponding to different applied cycles are illustrated in Figure 13a, in which the proportion of matrix cracking mode 3 is *η* = 0.2. The experimental and theoretical fatigue hysteresis dissipated energy as a function of interface shear stress is shown in Figure 13b. The theoretical fatigue hysteresis dissipated energy increases with decreasing interface shear stress to the peak value of 32.1 kJ/m^3^, and then decreases with decreasing interface shear stress to 0 kJ/m^3^. The experimental fatigue hysteresis dissipated energy of the 121st and 1,200,331st applied cycles lie in the right part of the fatigue hysteresis dissipated energy versus interface shear stress curve. The fatigue hysteresis loops correspond to interface slip Case 2, i.e., the interface partially debonds, and fiber slips partially relative to matrix in the interface debonded region. With the number of applied cycles increasing, interface shear stress decreases with increasing cycle number due to interface wear and interface oxidation. By comparing experimental fatigue hysteresis dissipated energy with theoretical values, the interface shear stress corresponding to different applied cycles applied cycles can be estimated, as shown in Table 8.

**Figure 14:**
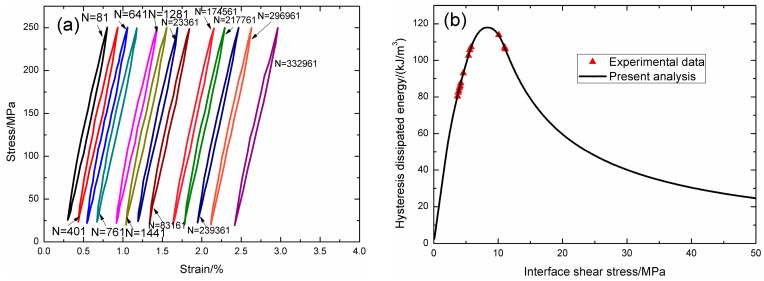
(**a**) The experimental fatigue hysteresis loops corresponding to different cycle number; and (**b**) the experimental and theoretical fatigue hysteresis dissipated energy versus interface shear stress curve of 2D woven SiC/SiC composite under *σ*_max_ = 250 MPa at room temperature.

Under fatigue peak stress of *σ*_max_ = 250 MPa, the fatigue hysteresis loops corresponding to different applied cycles are illustrated in Figure 14a, in which the proportion of matrix cracking mode 3 is *η* = 0.8. The experimental and theoretical fatigue hysteresis dissipated energy as a function of interface shear stress is shown in Figure 14b. The theoretical fatigue hysteresis dissipated energy increases with decreasing interface shear stress to the peak value of 118 kJ/m^3^, and then decreases with decreasing interface shear stress to 0 kJ/m^3^. The experimental fatigue hysteresis dissipated energy of the 81st, 401st, and 641st applied cycles lie in the right part of the fatigue hysteresis dissipated energy versus interface shear stress curve. The fatigue hysteresis loops correspond to interface slip Case 2, i.e., the interface partially debonds, and fiber slips partially relative to matrix in the interface debonded region. With the number of applied cycles increasing, interface shear stress decreases with increasing cycle number due to interface wear and interface oxidation. By comparing experimental fatigue hysteresis dissipated energy with theoretical values, the interface shear stress corresponding to different applied cycles can be estimated, as shown in Table 9. The fatigue hysteresis loops of the 761st and 332,961st applied cycles correspond to interface slip Case 4, i.e., the interface completely debonds, and fiber slips completely relative to matrix in the interface debonded region.

#### 4.2.2. Elevated Temperature

Shi [11] investigated the tension–tension fatigue behavior of 2D woven SiC/SiC composite at 800 °C in air. The fatigue loading was in a sinusoidal waveform and a loading frequency of 1.0 Hz, and the fatigue stress ratio was 0.1. The material properties of 2D woven SiC/SiC composite are given by: *V*_f_ = 40%, *E*_f_ = 270 GPa, *E*_m_ = 300 GPa, *r*_f_ = 7.0 μm, *α*_f_ = 5.1 × 10^−6^/°C, *α*_m_ = 4.7 × 10^−6^/°C, and Δ*T* = −200 °C.

**Figure 15:**
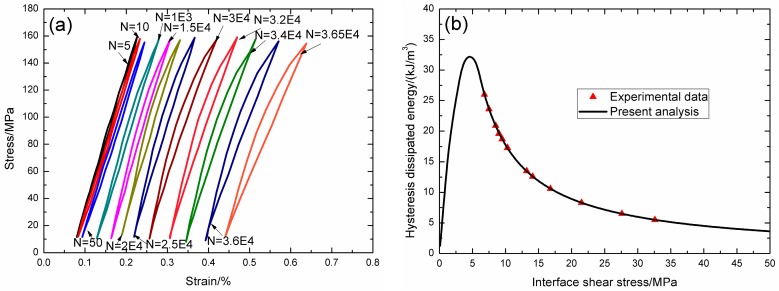
(**a**) The experimental fatigue hysteresis loops corresponding to different cycle number; and (**b**) the experimental and theoretical fatigue hysteresis dissipated energy versus interface shear stress curve of 2D woven SiC/SiC composite under *σ*_max_ = 150 MPa at 800 °C in air.

Under fatigue peak stress of *σ*_max_ = 150 MPa, the fatigue hysteresis loops corresponding to different applied cycles are illustrated in Figure 15a, in which the proportion of matrix cracking mode 3 is *η* = 0.2. The experimental and theoretical fatigue hysteresis dissipated energy as a function of interface shear stress is shown in Figure 15b. The theoretical fatigue hysteresis dissipated energy increases with decreasing interface shear stress to the peak value of 32.1 kJ/m^3^, and then decreases with decreasing interface shear stress to 0 kJ/m^3^. The experimental fatigue hysteresis dissipated energy of the 5th and 36,500th applied cycles lie in the right part of the fatigue hysteresis dissipated energy versus interface shear stress curve. The fatigue hysteresis loops correspond to interface slip Case 2, i.e., the interface partially debonds, and fiber slips partially relative to matrix in the interface debonded region. With the number of applied cycles increasing, interface shear stress decreases with increasing cycle number due to interface wear and interface oxidation. By comparing experimental fatigue hysteresis dissipated energy with theoretical values, the interface shear stress corresponding to different applied cycles can be estimated, as shown in Table 10.

Groner [12] investigated the tension–tension fatigue behavior of 2D woven SiC/SiC composite at 1100 °C in air. The Nicalon™ SiC (Nippon Carbon Co., Ltd., Tokyo, Japan) fiber-reinforced silicon carbide matrix composites (SiC/SiC CMCs) were manufactured by chemical vapor infiltration (CVI) method. The fatigue loading was in a triangular waveform and a loading frequency of 1.0 Hz, and the fatigue stress ratio was 0.1. The material properties of 2D woven SiC/SiC composite are given by: *V*_f_ = 40%, *E*_f_ = 230 GPa, *E*_m_ = 350 GPa, *r*_f_ = 7.0 μm, *α*_f_ = 3.9 × 10^−6^/K, *α*_m_ = 2 × 10^−6^/K, and Δ*T* = −100 K.

**Figure 16:**
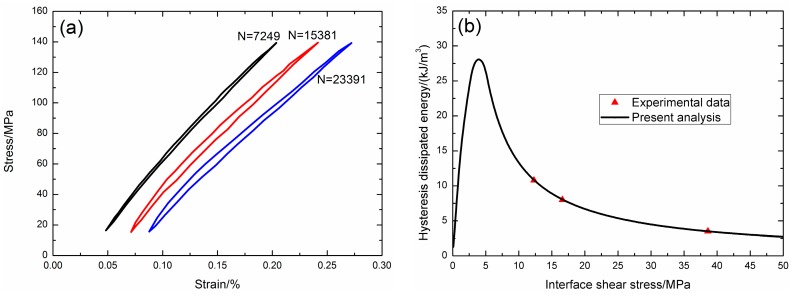
(**a**) The experimental fatigue hysteresis loops corresponding to different cycle number; and (**b**) the experimental and theoretical fatigue hysteresis dissipated energy versus interface shear stress curve of 2D woven SiC/SiC composite under *σ*_max_ = 140 MPa at 1100 °C in air.

Under fatigue peak stress of *σ*_max_ = 140 MPa, the fatigue hysteresis loops corresponding to different applied cycles are illustrated in Figure 16a, in which the proportion of matrix cracking mode 3 is *η* = 0.2. The experimental and theoretical fatigue hysteresis dissipated energy as a function of interface shear stress is shown in Figure 16b. The theoretical fatigue hysteresis dissipated energy increases with decreasing interface shear stress to the peak value of 28 kJ/m^3^, and then decreases with decreasing interface shear stress to 0 kJ/m^3^. The experimental fatigue hysteresis dissipated energy of the 7249th, 15,381st, and 23,391st applied cycles all lie in the right part of the fatigue hysteresis dissipated energy versus interface shear stress curve. The fatigue hysteresis loops correspond to interface slip Case 2, i.e., the interface partially debonds, and fiber slips partially relative to matrix in the interface debonded region. With the number of applied cycles increasing, interface shear stress decreases with increasing cycle number due to interface wear and interface oxidation. By comparing the experimental fatigue hysteresis dissipated energy with theoretical values, the interface shear stress corresponding to different applied cycles can be estimated, as shown in Table 11.

### 4.3. Comparison Analysis

**Figure 17:**
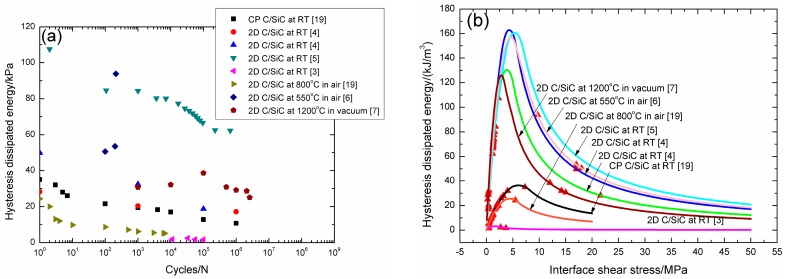
(**a**) The experimental fatigue hysteresis dissipated energy versus applied cycles; and (**b**) the experimental and theoretical fatigue hysteresis dissipated energy versus interface shear stress curves of cross-ply and 2D woven C/SiC composites at room and elevated temperatures.

The experimental fatigue hysteresis dissipated energy versus cycle number curves of cross-ply and 2D woven C/SiC composites at room temperature, 550 °C, and 800 °C in air, and 1200 °C in vacuum are illustrated in Figure 17a. For cross-ply C/SiC composite under *σ*_max_ = 105 MPa at room temperature and 800 °C in air, 2D woven C/SiC composite under *σ*_max_ = 335 and 375 MPa at room temperature, the experimental fatigue hysteresis dissipated energy decreases with increasing cycle number, and lies in the right and left part of the fatigue hysteresis dissipated energy versus interface shear stress curve, as shown in Figure 17b. For 2D woven C/SiC composite under *σ*_max_ = 350 MPa at 550 °C in air, the experimental fatigue hysteresis dissipated energy increases with increasing cycle number, and lies in the right part of the fatigue hysteresis dissipated energy versus interface shear stress curve, as shown in Figure 17b. For 2D woven C/SiC composite under *σ*_max_ = 57 MPa at room temperature, and *σ*_max_ = 300 MPa at 1200 °C in vacuum, the experimental fatigue hysteresis dissipated energy increases first, and then decreases with increasing cycle number, and lies in the right and left part of the fatigue hysteresis dissipated energy versus interface shear stress curve, as shown in Figure 17b.

The interface shear stress degradation rate of cross-ply and 2D woven C/SiC composites at room and elevated temperatures are illustrated in Table 12. The interface shear stress degradation rate is the highest for 2D woven C/SiC composite under *σ*_max_ = 350 MPa at 550 °C in air, i.e., 7.4 × 10^−2^ MPa/cycle, due to high fatigue peak stress and low loading frequency of 0.1 Hz, and is the lowest for 2D woven C/SiC composite under *σ*_max_ = 375 MPa at room temperature, i.e., 1.7 × 10^−6^ MPa/cycle, due to high loading frequency of 40 Hz. For cross-ply C/SiC composite under the same fatigue peak stress of *σ*_max_ = 105 MPa at room temperature and 800 °C in air, the interface shear stress degradation rate increases at elevated temperature in air, i.e., 7.7 × 10^−4^ MPa/cycle at elevated temperature and 6.3 × 10^−6^ MPa/cycle at room temperature, due to interface oxidation and interface wear. For 2D woven C/SiC composite at room temperature, the interface shear stress degradation rate increases with increasing fatigue peak stress, i.e., 2.1 × 10^−5^ MPa/cycle under *σ*_max_ = 335 MPa, and 1.6 × 10^−4^ MPa/cycle under *σ*_max_ = 375 MPa, and decreases with increasing loading frequency, i.e., 1.6 × 10^−4^ MPa/cycle at the loading frequency of 10 Hz, and 1.7 × 10^−6^ MPa/cycle at the loading frequency of 40 Hz under the same fatigue peak stress of *σ*_max_ = 375 MPa.

**Figure 18:**
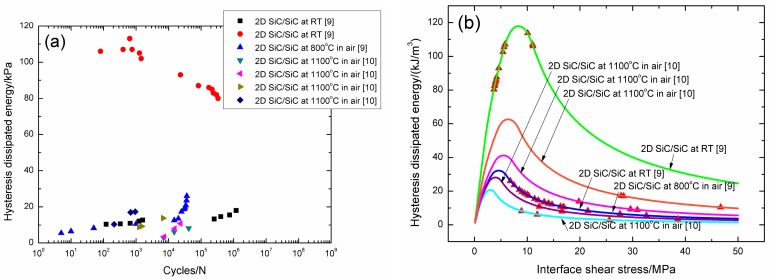
(**a**) The experimental fatigue hysteresis dissipated energy versus applied cycles; and (**b**) the experimental and theoretical fatigue hysteresis dissipated energy versus interface shear stress curves of 2D woven SiC/SiC composites at room and elevated temperatures.

The experimental fatigue hysteresis dissipated energy versus cycle number curves of 2D SiC/SiC composite at room temperature, 800 °C, and 1100 °C in air are illustrated in Figure 18a. For 2D SiC/SiC composite under *σ*_max_ = 150 MPa at room temperature and 800 °C in air, and under *σ*_max_ = 120, 140, 170, and 210 MPa at 1100 °C in air, the experimental fatigue hysteresis dissipated energy increases with increasing cycle number, and lies in the right part of the fatigue hysteresis dissipated energy versus interface shear stress curve, as shown in Figure 18b. The experimental fatigue hysteresis dissipated energy first increases, and then decreases with increasing cycle number, and lies in the right and left part of the fatigue hysteresis dissipated energy versus interface shear stress curve, corresponding to the fatigue loading of 2D SiC/SiC composite under *σ*_max_ = 250 MPa at room temperature, as shown in Figure 18a,b, respectively.

The interface shear stress degradation rate of 2D woven SiC/SiC composite at room and elevated temperatures are illustrated in Table 12. The interface shear stress degradation rate is the highest for 2D woven SiC/SiC composite under *σ*_max_ = 210 MPa at 1100 °C in air, i.e., 2.6 × 10^−2^ MPa/cycle, and the lowest for 2D woven SiC/SiC composite under *σ*_max_ = 150 MPa at room temperature, i.e., 5.8 × 10^−6^ MPa/cycle. For 2D SiC/SiC composite under the same fatigue peak stress of *σ*_max_ = 150 MPa at room temperature and 800 °C in air, the interface shear stress degradation rate increases at elevated temperature in air, i.e., 7.0 × 10^−4^ MPa/cycle at elevated temperature, and 5.8 × 10^−6^ MPa/cycle at room temperature, due to interface oxidation and interface wear. For 2D woven SiC/SiC composite at room temperature, the interface shear stress degradation rate increases with increasing fatigue peak stress, i.e., 5.8 × 10^−6^ MPa/cycle under *σ*_max_ = 150 MPa, and 2.2 × 10^−5^ MPa/cycle under *σ*_max_ = 250 MPa. For 2D woven SiC/SiC composite at 1100 °C in air, the interface shear stress degradation rate increases with increasing fatigue peak stress, i.e., 4.8 × 10^−4^ MPa/cycle under *σ*_max_ = 120 MPa, and 2.6 × 10^−2^ MPa/cycle under *σ*_max_ = 210 MPa.

**Figure 19:**
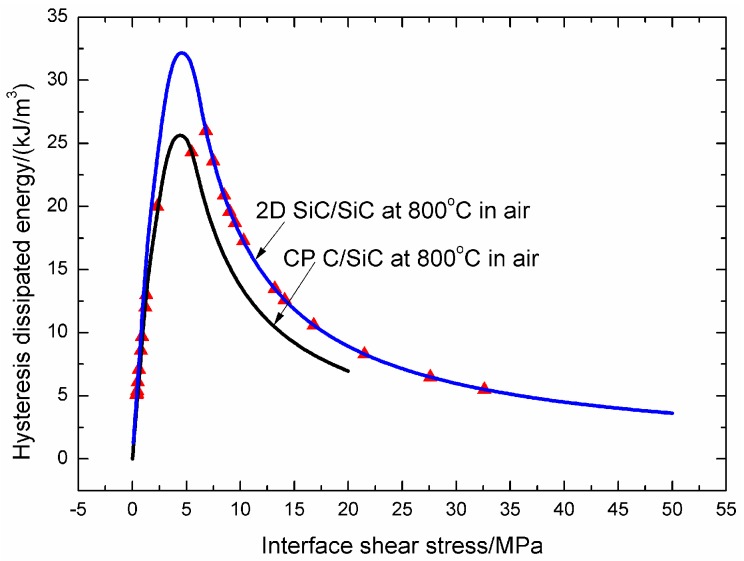
The experimental and theoretical fatigue hysteresis dissipated energy of cross-ply C/SiC and 2D woven SiC/SiC composites at 800 °C in air.

The experimental and theoretical fatigue hysteresis dissipated energy versus interface shear stress curves of cross-ply C/SiC and 2D woven SiC/SiC composites at 800 °C in air are illustrated in Figure 19. The experimental fatigue hysteresis dissipated energy of cross-ply C/SiC composite lies in the right and left part of the fatigue hysteresis dissipated energy versus interface shear stress curve, corresponding to interface slip Case 2 and Case 4, i.e., the interface partially debonds and fiber slips partially slips relative to matrix, and the interface completely debonds and fiber slips completely relative to matrix; however, for 2D woven SiC/SiC composite, the experimental fatigue hysteresis dissipated energy just lies in the right part of the fatigue hysteresis dissipated energy versus interface shear stress curve, corresponding to interface slip Case 2, i.e., the interface partially debonds and fiber slips partially relative to matrix. At room temperature and 800 °C in air, the interface shear stress degradation rate of 2D woven SiC/SiC composite under *σ*_max_ = 150 MPa is lower, i.e., 5.8 × 10^−6^ MPa/cycle at room temperature, and 7.0 × 10^−4^ MPa/cycle at 800 °C in air, than that of cross-ply C/SiC composite under *σ*_max_ = 105 MPa, i.e., 6.3 × 10^−6^ MPa/cycle at room temperature, and 7.7 × 10^−4^ MPa/cycle at 800 °C in air.

## 5. Conclusions

The comparisons of damage evolution between 2D C/SiC and SiC/SiC composites under tension–tension cyclic fatigue loading at room and elevated temperatures have been investigated. The fatigue hysteresis loops models considering multiple matrix cracking modes in 2D CMCs have been developed based on the damage mechanism of fiber slipping relative to matrix in the interface debonded region. The relationships between fatigue hysteresis loops, fatigue hysteresis dissipated energy, fatigue peak stress, matrix multiple cracking modes, and interface shear stress have been established. The effects of fiber volume fraction, fatigue peak stress and matrix cracking mode proportion on fatigue hysteresis dissipated energy and interface debonding and slipping have been analyzed. The experimental fatigue hysteresis dissipated energy of 2D C/SiC and SiC/SiC composites at room temperature, 550 °C, 800 °C, and 1100 °C in air, 1200 °C in vacuum corresponding to different fatigue peak stresses and cycle numbers have been analyzed. The interface shear stress degradation rate has been obtained through comparing experimental fatigue hysteresis dissipated energy with theoretical values. The damage evolution in C/SiC and SiC/SiC composites has been compared using damage parameters of fatigue hysteresis dissipated energy and interface shear stress degradation rate.

1. The interface shear stress degradation rate increases at elevated temperature in air compared with that at room temperature due to interface and fiber oxidation, decreases with increasing loading frequency at room temperature due to the increasing interface wear rate between fibers and the matrix, and increases with increasing fatigue peak stress at room and elevated temperatures due to the increasing interface debonding and slipping extent between fibers and the matrix.
2. At 800 °C in air, the experimental fatigue hysteresis dissipated energy of cross-ply C/SiC composite under *σ*_max_ = 105 MPa lies in the right and left part of the fatigue hysteresis dissipated energy versus interface shear stress curve, corresponding to interface slip Case 2 and Case 4, which indicates that the interface debonding changes from partially debonding to completely debonding with increasing cycle number; however, for 2D woven SiC/SiC composite under *σ*_max_ = 150 MPa, the experimental fatigue hysteresis dissipated energy just lies in the right part of the fatigue hysteresis dissipated energy versus interface shear stress curve, corresponding to interface slip Case 2, which indicates that the interface debonding remains to be partially debonding with increasing cycle number.
3. At room temperature and 800 °C in air, the interface shear stress degradation rate of 2D woven SiC/SiC composite under *σ*_max_ = 150 MPa is lower than that of cross-ply C/SiC composite under *σ*_max_ = 105 MPa.

In the present analysis, the effect of mechanical load waveform, i.e., sinusoidal waveform and triangular waveform, on fatigue damage in C/SiC and SiC/SiC composites has not been considered [24], which would be further investigated in the future.

## Figures and Tables

**Table 1:** The interface shear stress of cross-ply C/SiC composite corresponding to different applied cycles under fatigue peak stress of *σ*_max_ = 105 MPa at room temperature.

Cycle Number	Experimental Hysteresis Dissipated Energy/(kJ/m^3^)	Interface Shear Stress/MPa
1	35	7.3
3	32	4.0
5	28	3.2
7	26	2.8
100	21.5	2.1
1000	19.4	1.9
4000	18.2	1.8
10,000	16.9	1.6
100,000	12.8	1.2
1,000,000	10.7	1.0

**Table 2:** The interface shear stress of 2D woven C/SiC composite corresponding to different applied cycles under fatigue peak stress of *σ*_max_ = 335 MPa at room temperature.

Cycle Number	Experimental Hysteresis Dissipated Energy/(kJ/m^3^)	Interface Shear Stress/MPa
1	28.2	22
1000	20.3	0.4
1,000,000	17.1	0.35

**Table 3:** The interface shear stress of 2D woven C/SiC composite corresponding to different applied cycles under fatigue peak stress of *σ*_max_ = 375 MPa at room temperature.

Cycle Number	Experimental Hysteresis Dissipated Energy/(kJ/m^3^)	Interface Shear Stress/MPa
2	107.5	2.3
107	84.6	1.7
998	84.3	1.68
3659	80.2	1.55
7124	80.1	1.54
16,931	77.3	1.48
26,982	74.6	1.4
36,408	73.3	1.38
45,961	71.9	1.32
56,119	70.6	1.31
66,277	69.2	1.3
78,275	67.9	1.28
95,575	66.6	1.2
227,112	62.5	1.15
659,419	62.3	1.14

**Table 4:** The interface shear stress of 2D woven C/SiC composite corresponding to different applied cycles under fatigue peak stress of *σ*_max_ = 57 MPa at room temperature.

Cycle Number	Experimental Hysteresis Dissipated Energy/(kJ/m^3^)	Interface Shear Stress/MPa
11,104	1.8	3.7
33,262	2.4	2.7
55,493	1.7	0.5
100,000	1.6	0.45

**Table 5:** The interface shear stress of cross-ply C/SiC composite corresponding to different applied cycles under fatigue peak stress of *σ*_max_ = 105 MPa at 800 °C in air.

Cycle Number	Experimental Hysteresis Dissipated Energy/(kJ/m^3^)	Interface Shear Stress/MPa
1	24.3	5.5
2	20	2.3
3	13	1.3
4	12	1.2
10	9.7	0.9
100	8.6	0.8
500	7.1	0.6
1000	6.1	0.5
3000	5.4	0.45
6000	5.2	0.43
6600	5.1	0.4

**Table 6:** The interface shear stress of 2D woven C/SiC composite corresponding to different applied cycles under fatigue peak stress of *σ*_max_ = 350 MPa at 550 °C in air.

Cycle Number	Experimental Hysteresis Dissipated Energy/(kJ/m^3^)	Interface Shear Stress/MPa
100	50.5	18
200	53.4	17
212	93.8	9.7

**Table 7:** The interface shear stress of 2D woven C/SiC composite corresponding to different applied cycles under fatigue peak stress of *σ*_max_ = 300 MPa at 1200 °C in vacuum.

Cycle Number	Experimental Hysteresis Dissipated Energy/(kJ/m^3^)	Interface Shear Stress/MPa
1000	30.7	15
10,000	32.1	14.2
100,000	38.6	12
500,000	30.8	0.4
1,000,000	29.1	0.35
2,100,000	28.6	0.3
2,600,000	25	0.25

**Table 8:** The interface shear stress of 2D woven SiC/SiC composite corresponding to different applied cycles under fatigue peak stress of *σ*_max_ = 150 MPa at room temperature.

Cycle Number	Experimental Hysteresis Dissipated Energy/(kJ/m^3^)	Interface Shear Stress/MPa
121	10.4	17
331	10.6	16.8
661	10.9	16.3
1241	12.1	14.7
1601	12.7	14
253,561	13.3	13.3
405,451	14.6	12.1
757,531	15.5	11.4
1,200,331	18	10

**Table 9:** The interface shear stress of 2D woven SiC/SiC composite corresponding to different applied cycles under fatigue peak stress of *σ*_max_ = 250 MPa at room temperature.

Cycle Number	Experimental Hysteresis Dissipated Energy/(kJ/m^3^)	Interface Shear Stress/MPa
81	106	11.1
401	107	11
641	113	10.1
761	107	5.8
1281	105	5.6
1441	102	5.4
23,361	93	4.6
83,161	87	4.2
174,561	86	4.1
217,761	85	4
239,361	83	3.9
296,961	82	3.8
332,961	80	3.7

**Table 10:** The interface shear stress of 2D woven SiC/SiC composite corresponding to different applied cycles under fatigue peak stress of *σ*_max_ = 150 MPa at 800 °C in air.

Cycle Number	Experimental Hysteresis Dissipated Energy/(kJ/m^3^)	Interface Shear Stress/MPa
5	5.5	32.6
10	6.5	27.6
50	8.3	21.5
1000	10.6	16.8
15,000	12.6	14.1
20,000	13.5	13.2
25,000	17.3	10.3
30,000	18.7	9.5
32,000	19.6	9
34,000	20.9	8.5
36,000	23.6	7.5
36,500	26	6.8

**Table 11:** The interface shear stress of 2D woven SiC/SiC composite corresponding to different applied cycles under fatigue peak stress of *σ*_max_ = 140 MPa at 1100 °C in air.

Cycle Number	Experimental Hysteresis Dissipated Energy/(kJ/m^3^)	Interface Shear Stress/MPa
7249	3.5	38.6
15,381	8	16.6
23,391	10.8	12.3

**Table 12:** The interface shear stress degradation rate of C/SiC and SiC/SiC composites at room and elevated temperatures.

Items	*T*	*σ*_max_/MPa	*τ*_initial_/MPa	*τ*_final_/MPa	*N*_initial_	*N*_final_	*ψ*/(MPa/Cycle)
CP C/SiC	RT	105	7.3	1	1	1,000,000	6.3 × 10^−6^
	800 °C in air	105	5.5	0.4	1	6600	7.7 × 10^−4^
2D C/SiC	RT	335	21.6	0.35	1	1,000,000	2.1 × 10^−5^
	RT	375	17	0.45	1	100,000	1.6 × 10^−4^
	RT	375	2.3	1.14	2	659,419	1.7 × 10^−6^
	RT	57	3.7	0.45	11,104	100,000	3.6 × 10^−5^
	550 °C in air	350	18	9.7	100	212	7.4 × 10^−2^
	1200 °C in vacuum	300	15	0.25	1000	2,600,000	5.6 × 10^−6^
2D SiC/SiC	RT	150	17	10	121	1,200,331	5.8 × 10^−6^
	RT	250	11.1	3.7	81	332,961	2.2 × 10^−5^
	800 °C in air	150	32.6	6.8	5	36,500	7.0 × 10^−4^
	1100 °C in air	120	25.6	8.9	7062	41,696	4.8 × 10^−4^
	1100 °C in air	140	38.6	12.3	7249	23,391	1.6 × 10^−3^
	1100 °C in air	170	30.9	19.8	1242	6780	2.0 × 10^−3^
	1100 °C in air	210	46.7	27.8	212	945	2.6 × 10^−2^
